# Left Ventricular Rupture Due to Congenital Partial Defect of the Left Ventricular Free Wall

**DOI:** 10.1093/icvts/ivag028

**Published:** 2026-01-27

**Authors:** Ryoichi Kondo, Rumi Haneda, Yoichiro Hirata, Kagami Miyaji

**Affiliations:** Department of Cardiovascular Surgery, Kitasato University, School of Medicine, Minami-ku, Kitasato, 1-15-1, Sagamihara, Kanagawa-ken, 252-0375, Japan; Department of Pediatrics, Kitasato University, School of Medicine, Minami-ku, Kitasato, 1-15-1, Sagamihara, Kanagawa-ken, 252-0375, Japan; Department of Pediatrics, Kitasato University, School of Medicine, Minami-ku, Kitasato, 1-15-1, Sagamihara, Kanagawa-ken, 252-0375, Japan; Department of Cardiovascular Surgery, Kitasato University, School of Medicine, Minami-ku, Kitasato, 1-15-1, Sagamihara, Kanagawa-ken, 252-0375, Japan

**Keywords:** left ventricular rupture, neonatal cardiac surgery, congenital defect, TAPVC, pseudoaneurysm

## Abstract

A neonate who underwent corrective surgery for cardiac-type total anomalous pulmonary venous connection (TAPVC) was suspected of having a pseudoaneurysm of the left ventricular (LV) free wall on transthoracic echocardiography (TTE) on postoperative day 11. Emergency surgery was performed the following day, revealing LV rupture due to a congenital partial defect of the LV free wall. The defect was successfully repaired using double-patch closure reinforced with BioGlue. The postoperative course was uneventful. This case highlights that left ventricular rupture may occur due to an unrecognized congenital defect after neonatal cardiac surgery, particularly in conditions such as TAPVC, where the left ventricle is underfilled preoperatively.

## CLINICAL CASE

LV free wall rupture is a known but fatal complication following cardiac surgery. In this case, although the rupture occurred postoperatively, suggesting a potential surgical aetiology, intraoperative findings strongly indicated that the rupture was due to a congenital partial defect of the LV free wall.

The patient was a full-term neonate born via spontaneous vaginal delivery at 39 weeks and 4 days of gestation, with a birth weight of 3138 g. No congenital heart disease was detected prenatally. However, a few hours after birth, the infant developed cyanosis and oxygenation failure, prompting transfer to a specialized center. TTE performed at the referral hospital confirmed a diagnosis of cardiac-type TAPVC (type IIa), and elective surgical repair was planned.

On day 21 of life, the patient underwent median sternotomy under cardiopulmonary bypass (CPB) and cardiac arrest. The coronary sinus was cut back, and rerouting was performed using an ePTFE patch to correct the TAPVC. No left ventricular vent was used. The total surgical time, CPB time, and aortic cross-clamp time were 172 min, 63 min, and 34 min, respectively. The intraoperative and early postoperative courses were uneventful.

**Figure 1.**
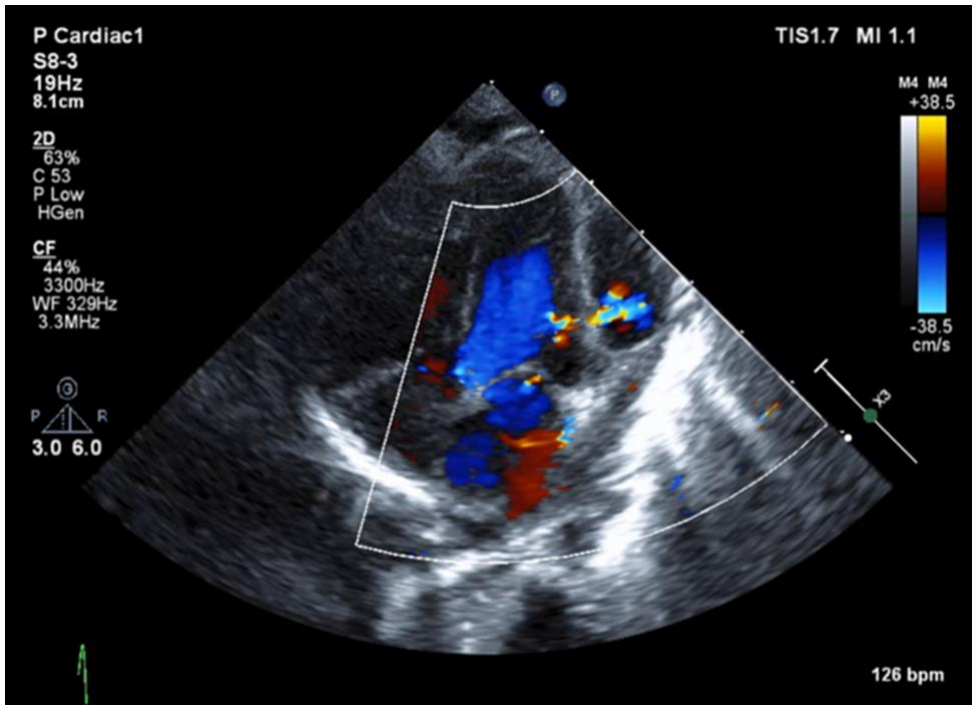
A Cavity is Observed on the Anterior Surface of the Left Ventricle, and Colour Doppler Imaging Reveals Blood Flow Entering from the Left Ventricle.

On postoperative day 10, prior to discharge, a routine TTE revealed an aneurysmal structure adjacent to the LV free wall. Colour Doppler imaging demonstrated communication with the LV, raising suspicion of a pseudoaneurysm (Figure 1). Contrast-enhanced CT performed the following day confirmed the presence of a pseudoaneurysm and a surrounding haematoma, suggesting impending rupture, necessitating emergency surgery.

**Figure 2.**
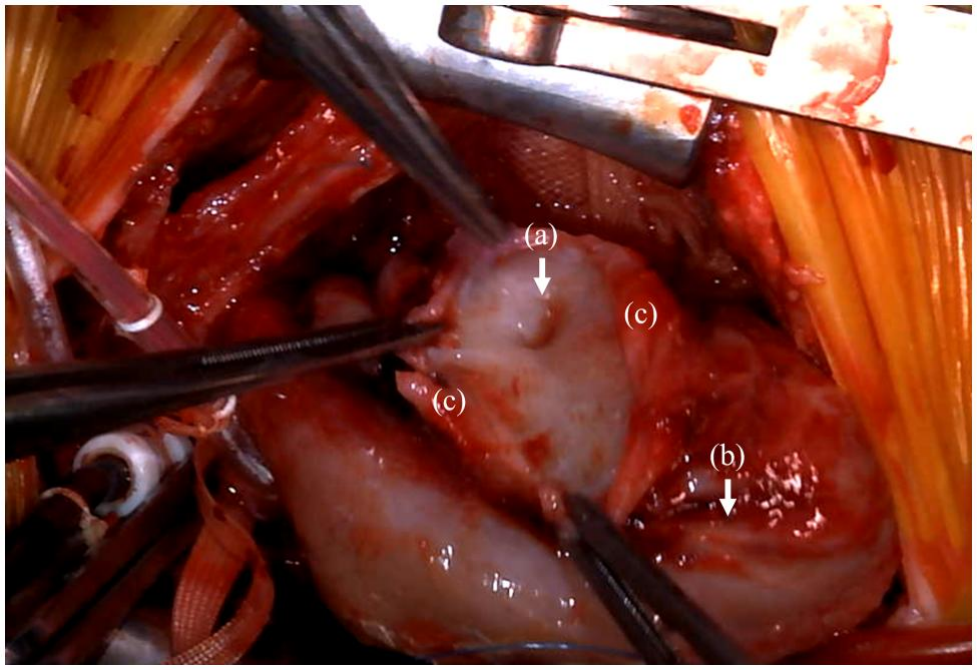
(A) A 4-5 mm Partial Defect of the Left Ventricular Myocardium is Observed Without Any Signs of Damage or Necrosis at the Margins, (B) Ruptured Epicardium, (C) Left Anterior Descending Artery.

The reoperation was performed on day 32 of life. Due to concerns about rupture upon re-entry, vascular access was secured in the right neck before reopening the sternum. A median resternotomy was performed with minimal dissection. CPB was initiated via ascending aortic perfusion and right atrial drainage, and a left heart vent was placed through the right-sided left atrium. After aortic cross-clamping and cardiac arrest, dissection was carried out over the anterior LV surface. A 4-5 mm defect was identified approximately 1 cm to the left of the left anterior descending artery (Figure 2). The edges of the defect showed no evidence of Haemorrhage, necrosis, or surgical injury, strongly suggesting a congenital partial defect of the LV free wall. Surrounding the defect, the epicardium was detached over an area of approximately 1 cm circumferentially and 2 cm caudally, indicating that the LV free wall defect had been covered by the epicardium, which later ruptured.

Initial attempts at direct closure using pledgeted polypropylene sutures resulted in myocardial tearing due to the fragile tissue. Consequently, bovine pericardium was sutured to the epicardium for closure, reinforced with BioGlue, and further secured with an additional bovine pericardial patch using a double-patch technique. After weaning from CPB, no bleeding was observed from the repair site. The total surgical time, CPB time, and aortic cross-clamp time were 262 min, 124 min, and 93 min, respectively.

The postoperative course was favourable. Postoperative TTE showed reduced wall motion of the LV free wall due to surgical manipulation, but overall LV contraction was preserved with an ejection fraction of 56%. No residual pseudoaneurysm was observed. Additionally, contrast-enhanced CT showed no evidence of pseudoaneurysm formation. The patient was discharged home on postoperative day 19.

## DISCUSSION

To date, only one other case of LV free wall rupture following neonatal cardiac surgery has been reported: Jaurena et al. described a case of LV free wall rupture 2 weeks after an arterial switch operation.^1^ In their case, coronary artery transfer and a postoperative requirement for extracorporeal membrane oxygenation for 3 days suggested that myocardial ischaemia was the underlying cause.

A condition known as left ventricular myocardial crypt (LVMC) is characterized by defects penetrating more than 50% of the myocardial wall. According to Sigvardsen et al., LVMC is considered a minor marker of hypertrophic cardiomyopathy but is also present in 9.1% of healthy individuals, with no significant association with major cardiovascular events.^2^ In their study, 95% of LVMCs were found in the posterior wall, but 3% were located in the anterior wall, where the rupture occurred in our case. LVMC has been reported more frequently in young individuals, those with hypotension, and those with preserved cardiac function.

In our patient, preoperatively, LV volume overload was minimal due to the nature of TAPVC, which may have resulted in a relatively small LV. If a congenital LV free wall defect had been present, it might have remained clinically silent due to epicardial coverage. However, after TAPV repair, LV volume load normalized, leading to epicardial dehiscence and subsequent LV rupture. Furthermore, the possibility that LVMC contributed to the congenital LV free wall defect cannot be ruled out. In cardiac-type TAPVC, the heart is often not lifted during the repair, which may allow a small congenital defect of the LV free wall to go unnoticed. This case suggests that similar LV ruptures could occur in other conditions where the LV is chronically underfilled and subsequently experiences an abrupt increase in volume load. Awareness of this mechanism is essential when evaluating postoperative LV rupture in neonates.

## Data Availability

Raw data were generated at Kitasato University Hospital. The derived data supporting the findings of this study are available from the corresponding author, Ryoichi Kondo, upon reasonable request.
